# 

**Published:** 1893-07

**Authors:** 


### CONTENTS:

Article I.—Sarcoma of Lower Jaw—Varicocele—Rarefying Ostitis of Tibia—Deformity of Lower Extremity—Angeio-Sarcoma of Testis. By Charles M'Burney, M. D. - - - - - - - - - 97

" II.—Removal Bridge or Plate Work. By Dr. Chas. L. Van Fossen. - - - - - - - 105

" III.—Unnecessary Pain in Dental Operations. By Dr. James A. Reilly. - - - - - - - - 110

" IV.—The Conservative Treatment of Teeth by Gold Process. By Francis Peabody. - - - - 113

" V.—An Act to Regulate the Practice of Dentistry within the Territory of New Mexico. - - - - - 120

" VI.—Immediate Root Filling. By Junius E. Cravens, D. D. S. - - - - - - - - - 123

" VII.—Anæsthetics. - - - - - - 129

" VIII.—Cholera Precautions. - - - - - 133

### EDITORIAL.

Progress in Therapeutics. - - - - - - - - 134

### MONTHLY SUMMARY.

A Difficult Case to Control. - - - - - - - 136

A Misplaced Canine. - - - - - - - - 137

Campho-phenique. - - - - - - - - - 138

Fermentation. - - - - - - - - - 139

Abscess of the Antrum of Highmore. - - - - - 140

Period of Infectiousness. - - - - - - - 141

Exemption of Dentists from Jury Duty. - - - - 142

Dr. Chas. B. Atkinson. - - - - - - - 142

Treatment of the Ear. - - - - - - - 143

A Hint. - - - - - - - - 143

Come to Our Arms. - - - - - - - - - 143

Antiseptic Properties of Tobacco. - - - - - 144

Soldering Aluminum. - - - - - - - - 144

